# Pregnancy Outcomes With COVID-19 Lessons Learned From the Pandemic

**DOI:** 10.7759/cureus.16358

**Published:** 2021-07-13

**Authors:** Saima Siddiqui, Rehana Najam

**Affiliations:** 1 Department of Obstetrics and Gynaecology, Teerthanker Mahaveer Medical College and Research Centre, Moradabad, IND

**Keywords:** sars cov-2, covid-19, maternal outcomes, pregnancy outcomes, vertical transmission, neonatal outcome, coronavirus, feto-maternal outcome

## Abstract

Background

Severe acute respiratory syndrome coronavirus-2 (SARS-CoV-2) that causes Coronavirus disease has caused one of the most damaging pandemics in the recorded human history.

Objective

To assess pregnancy outcomes with COVID-19 lessons learned from the pandemic.

Study design

This retrospective observational study was conducted at Teerthanker Mahaveer Medical College and Research Centre, Moradabad, a level 3 COVID hospital in Northern India, with a patient pool of all the antenatal females diagnosed COVID 19 positive via a positive quantitative reverse transcriptase-polymerase chain reaction (qRT-PCR) test of maternal pharyngeal and nasal swab samples in the given time period of three months and ten days, i.e., May 25, 2020 to September 3, 2020. In conjunction with maternal outcomes, neonatal outcomes including evidence of perinatal transmission of SARS-CoV-2 was assessed by testing neonatal pharyngeal swab samples.

Results

Out of 100 COVID-19 positive patients, the average age of women was 26.2 years, 73 women (73%) were asymptomatic, and 50 patients (50%) women had associated co-morbidities such as anaemia in 38 (38%) women, gestational diabetes mellitus (GDM) and pregnancy-induced hypertension (PIH) in four patients (4%) each, respectively. No case of spontaneous abortion in early gestation was reported. Out of 100 patients, 32 (32%) patients delivered during their stay, out of which 17 women (53.1%) delivered via cesarean section which was performed mainly due to obstetric indications. One maternal death was reported due to antepartum eclampsia which was unrelated to COVID-19 complications. Five neonates were born prematurely, out of which three were delivered followed by spontaneous premature preterm rupture of membranes (PPROM).

The Appearance Pulse Grimace Activity Respiration (APGAR) score was recorded to be ≥9 at the five minutes mark in 28 out of 30 live babies (93.3%) and the birth weight of the babies ranged from 1.8 to 3.5 kg) with an average birth weight of 2.71 kg. Two neonatal deaths were reported due to respiratory distress. There were two documented intrauterine demise (IUD) cases both due to PIH. Furthermore, all 30 live neonates tested for SARS CoV-2 had negative results.

Conclusion

The spectrum of Coronavirus infection leans more towards asymptomatic and mild symptomatic clinical presentation. Favourably, the likelihood of spontaneous preterm birth was not escalated in our current study and remained low. The rate of intrauterine fetal demise and neonatal death were less. As none of the neonates tested positive for COVID-19, there is no corroborative proof of vertical perinatal transmission.

## Introduction

Severe acute respiratory syndrome coronavirus-2 (SARS-CoV-2) which causes Coronavirus disease has caused one of the most damaging pandemics in recorded human history [1]. True to its name, the COVID-19 pandemic was first accounted for in December 2019, in Wuhan, China, and the number of positive patients is increasing swiftly across the whole world. As of September 3, 2020, the total confirmed cases of COVID-19 around the world stand at 25,842,652 and have already caused 858,629 deaths all across the world [2].

According to the Indian Council of Medical Research (ICMR), the diagnosis of COVID-19 infection is a five-pillar structure with the pillars being namely, epidemiological vulnerability and exposure, clinical attributes, laboratory parameters, CT chest findings, and a positive qRT-PCR analysis of nasal and pharyngeal specimens [3].

Pregnancy being a unique physiological and immunological state makes child-bearing females more vulnerable to infections. Due to the increased amount of progesterone and estrogen during pregnancy, the upper respiratory tract becomes inflamed and because of upward shifting of the diaphragm due to the overgrowing uterus, there is also restricted expansion of lungs and therefore all of these factors lead to increased susceptibility to respiratory pathogens. Also, there are concerns owing to the potential results on fetal and neonatal outcomes apart from the consequences of COVID-19 on a child-bearing woman [4].

In 2009, pregnant women constituted only 1% of all patients infected by the influenza A subtype H1N1 virus, however, they comprised 5% of the total deaths caused by the H1N1 virus [5]. Likewise, SARS-CoV-2 and Middle Eastern respiratory syndrome (MERS-COV), during their respective outbreaks showed severe complications during pregnancy, leading to admission to intensive care units, endotracheal intubations, renal failure, and ultimately death [6,7].

Considering the impact of previous outbreaks on pregnant women, we realize the pressing need for specialized research and studies to help us figure out the bearing of COVID-19 both short term and long term on a pregnant female as well as her future offspring as the available data are scarce.

## Materials and methods

This is a retrospective observational study of prospectively collected data of patients admitted in the Department of Obstetrics and Gynaecology at Teerthanker Mahaveer Medical College and Research Center, Moradabad, a level 3 designated COVID hospital in northern India.

According to the Indian Council of Medical Research guidelines, all antenatal women from red-zone/hot-spot districts were tested for COVID-19 via reverse transcriptase-polymerase chain reaction (RT-PCR) test even if asymptomatic. The samples were collected either on an outpatient basis or after admission, in accordance with Ministry of Health and Family Welfare, Government of India guidelines.

All the antenatal patients who came out to be COVID-19 positive diagnosed via a positive quantitative qRT-PCR test of maternal pharyngeal and nasal swab samples according to ICMR guidelines were included in the study and were admitted at the centre.

The aim and objective of the current study were to assess the effect and outcome of COVID-19 on maternal and neonatal morbidity and mortality. For the aggregation of clinical data such as age, gestational age, parity, address, antenatal history, associated co-morbidities, history of contact/travel, any positive symptoms regarding coronavirus infection were noted in the triage area and the patients were admitted to COVID isolation wards, pre-labor room or to intensive care unit on the basis of their chief complaints and severity of the disease while maintaining all infection prevention and control measures with proper distancing between beds and maintaining proper hand-hygiene and positive sanitary habits. Keeping in mind the infective potential of the female, written and informed consent was relinquished and verbal consent was acquired from the patients and their attendants.

COVID-19 positive patients who were kept in isolation wards and pre-labor rooms were investigated according to the guideline and were prescribed immune-boosters and routine antenatal medications, while symptomatic patients were managed symptomatically in conjunction with the physician and pulmonologist. Asymptomatic patients were discharged according to ICMR guidelines at the time and advised additional strict home quarantine.

After delivery of the baby, the necessary information such as gestational age at the time of delivery, mode of delivery and outcome, cause of cesarean section (if applicable), birth weight of the baby, Appearance Pulse Grimace Activity and Respiration (APGAR) score, need of neonatal intensive care unit (NICU) admission was noted. Neonatal pharyngeal and nasal swabs were sent within 24 hours of birth and the result was taken into consideration. Direct breastfeeding while taking all necessary precautions like wearing an N95 mask, gloves, and hand-washing, and use of sanitizer was actively encouraged in such patients.

Data of 100 COVID-19-positive antenatal women admitted to the hospital within the study period of three months and ten days (May 25, 2020 to September 3, 2020) was retrospectively entered in a Microsoft Excel sheet. Continuous variables are expressed as mean ± standard deviation (SD). All categorical variables are expressed as frequency and percentages.

## Results

Demographic data

Table 1 shows the demographic outlook of the included patients. A maximum number of the patients were in the age group of 20-25 years. The mean age was 26.17±3.73 years; 63 women (63%) were multigravida. Majority of women presented in their third trimester. Approximately, two-thirds of patients, i.e., 35 antenatal women (35%) belonged to the upper-middle-class according to the Modified Kuppuswamy scale.

**Table 1:** Demographic Data of the Patients.

Parameters	Number of cases (n)	Percentage (%)
The age group of the patient (years)		
≤19	2	2
20-25	47	47
26-30	39	39
31-35	10	10
>35	2	2
Total	100	100
Socio-economic data (Modified Kuppuswamy scale)		
Upper class (26-29)	25	25
Upper middle (16-25)	35	35
Lower middle (11-15)	18	18
Upper lower (5-10)	16	16
Lower (below 5)	6	6
Total	100	100
Parity		
Primigravida	37	37
Multigravida	63	63
Total	100	100
Gestational age (weeks)		
>37 weeks	38	38
34-37 weeks	21	21
28-34 weeks	15	15
<28 weeks	26	26
Total	100	100

Maternal clinical presentation and co-morbidities

Table 2 shows that 73 women (73%) were asymptomatic positive without any appearance of symptoms in the period during their stay. Among the 27 symptomatic patients, cough, fever, myalgia, and sore throat were the most common symptoms being 55.5%, 37%, 37%, and 18.5%, respectively. Almost all the patients recovered within two weeks of admission except one patient who required intubation due to antepartum eclampsia. No other patient required oxygen support or ICU admission due to COVID-19 infection. No death was reported due to COVID-19 pneumonia, whereas one patient expired due to an obstetrical cause. As many as 50% of patients had comorbidities. Anaemia, hypothyroidism, and GDM were the most common co-morbidities 38%, 10%, and 4%, respectively, as shown in Table 2. Out of 38 anaemic patients, 11 required blood transfusion.

**Table 2:** Maternal Clinical Presentation and Co-Morbidities. DM: diabetes mellitus, GDM: gestational diabetes mellitus, PIH: pregnancy-induced hypertension.

Parameters	Cases (n)	Percentage (%)
Clinical presentation of the patient		
Asymptomatic	73	73
Symptomatic	27	27
Total	100	100
Symptoms n=27		
Fever	10	37
Sore throat	5	18.5
Myalgia	10	37
Diarrhoea	1	3.7
Cough	15	55.5
Recovery of the patients		
Within 15 days	99	99
>15 days	0	0
Expired	1	1
Maternal co-morbidities		
DM/GDM	4	4
Hypertension/PIH/eclampsia	4	4
Hypothyroidism	10	10
Anaemia	38	38
Hepatitis C	1	1

Maternal outcomes

Out of 100 patients, 32 (32%) patients got delivered during their stay at the hospital. The number of patients delivered by cesarean section were more, i.e., 53.1% as compared to vaginal delivery. Cesarean section was done only for an obstetrical indication as shown in Table 3. There were no spontaneous abortions or fetal loss reported. One baby was born before 34 completed weeks via emergency cesarean section in view of previous cesarean section in labour at 33 weeks whereas four babies were born before 37 completed weeks, three vaginal delivery following premature preterm rupture of membrane (PPROM), and one emergency cesarean section due to foetal distress.

**Table 3:** Maternal Outcomes. APH: antepartum haemorrhage, PIH: pregnancy-induced hypertension, HELLP syndrome: hemolysis-elevated liver enzymes-low platelet count syndrome, PPROM: premature preterm rupture of membranes, LSCS: lower segment cesarean section.

Parameters	Cases (n)	Percentage (%)
Mode of delivery, n=32		
Vaginal delivery	15	46.9
Cesarean section	17	53.1
Total	32	100
Indication of cesarean section, n=17		
Previous LSCS in labour	9	52.9
Fetal distress	5	29.5
APH	1	5.8
PIH/eclampsia/HELLP syndrome	2	11.8
COVID-19 pneumonia	0	0
Total	17	100
Gestational age at delivery		
Preterm birth before 34 weeks	1	3.1
Preterm birth before 34 weeks with spontaneous labor-PPROM	0	0
Preterm birth before 37 weeks	1	3.1
Preterm birth before 37 weeks with spontaneous labor-PPROM	3	9.4
Birth after 37 weeks	27	84.4
Total	32	100

Neonatal outcomes and complications

Table 4 shows neonatal outcome and complications in the baby; out of 32 babies delivered, two (6.3%) were documented intrauterine death (IUD) at the third trimester; the birth weight of the babies ranged from 1.8 to 3.5 kg with an average birth weight of 2.71 kg; 23 (71.9%) babies belonged to a normal weight range of 2.5-3.5 kg, whereas eight (26.7%) were low-birth-weight, i.e., <2.5 kg. APGAR score was within normal limits in most of the babies. Low APGAR was observed in two (6.7%) out of 30 babies (excluding two IUD), five (15.6%) babies were born preterm and 12 babies (40%) got admitted to NICU. All the babies tested COVID-19 negative implicating that there was no vertical transmission. Two babies expired due to respiratory distress.

**Table 4:** Neonatal Outcomes and Complications. IUD: intrauterine death, NICU: neonatal intensive care unit.

Parameters	Cases (n)	Percentage (%)
Neonatal outcome		
Live term birth	25	78.1
Live preterm birth	5	15.6
IUD	2	6.3
Total	32	100
Weight at birth (kg)		
<2.5 kg	8	26.7
2.5-3 kg	14	46.6
3.1-3.5 kg	8	26.7
>3.5 kg	0	0
Total	30	100
APGAR score (At 1 min)		
7-10	28	93.3
4-6	2	6.7
0-3	0	0
Total	30	100
Neonatal complications		
Baby in NICU	12	40
Cause of NICU admission, n=12		
Low birth weight	9	75
Respiratory distress	2	16.7
Meconium stained liquor	1	8.3
Neonatal deaths	2	6.7
COVID 19 positive	0	0
Total live births	30	100

## Discussion

Pneumonia is the most ubiquitous non-obstetric infectious condition that transpires during pregnancy. Viral pneumonia has higher rates of maternal as well as neonatal morbidity and mortality as compared to other bacterial etiologies [8]. History has taught us that the aftermath of viral illness in the past like H1N1, SARS-CoV, MERS pandemic, and Ebola outbreak has not been great for pregnant females causing various negative obstetrical outcomes including PPROM, preterm labour, intrauterine fetal death, and neonatal death [9-11].

Our current study reported that the majority of women around 74% acquired infection in their third trimester and belonged to the age group of 20-30 years of age. Studies like Liu et al. [12] and Fan et al. [13] concluded that the majority of patients in their third trimesters acquired infection and the median age was 30 years, similar to our study.

According to the Federation of Obstetrics and Gynaecological Society of India (FOGSI), the majority of people (pregnant and general population) may be asymptomatic or present with mild respiratory symptoms of COVID-19 infection [14]. As reported by the World Health Organisation (WHO), pregnant women having co-morbid conditions such as diabetes, hypertension, obesity, and associated medical risk factors may present with pneumonia and marked hypoxia or may progress rapidly to this state [15].

Most of the patients in the current study, around 73% were asymptomatic, and approximately 50% of patients had associated co-morbidities as anaemia, hypothyroidism, gestational diabetes mellitus (GDM), and pregnancy-induced hypertension (PIH). The current study reported cough, fever, and myalgia as most common symptoms in the pregnant females in 55.5%, 37%, and 37% of patients, respectively; similar to our study, fever, and cough were reported as the most common clinical manifestation in nine antenatal laboratory-confirmed COVID-19 positive women in their third trimester by Chen et al. [16], whereas sore throat, malaise, myalgia, diarrhoea, and difficulty in breathing were few of the other symptoms reported in the study.

In a study conducted by Nayak et al. [17] on 141 patients, abortion was seen in 4.25% of patients. In the present study, out of 22 patients presenting in their first and second trimesters, no spontaneous pregnancy loss was reported. In our present study of 100 patients, 32 patients delivered during their stay, out of which 53.1% delivered via cesarean section. Most studies suggest that most women underwent cesarean section [13,16,18]. Recent data tend to incline towards cesarean section; however, the ultimate approach of intrapartum management depends on the woman’s co-morbidities.

In the current study, a cesarean section was performed due to obstetric indications like the previous cesarean with scar tenderness, fetal distress, etc., and not because of pulmonary insult caused by COVID-19 pneumonia. While the health of the mother remains a priority, there is another major concern that arises in such cases, that being the impact of SARS CoV-2 infection on the neonate and its vertical transmission potential. In the study by Chen et al. [16], no fetal death, neonatal death, or neonatal asphyxia was reported which was conducted on nine pregnant females. Although there were reports of four neonates being born prematurely, they were not related to the COVID-19 infection. The above result is similar to our current study where five neonates were born prematurely, three followed by spontaneous PPROM.

The APGAR was recorded to be ≥9 at the five-minute mark in 28 out of 30 live babies (93.3%) and 71.9% of babies have a normal weight between 2.5 and 3.5 kg. Two neonatal deaths were reported due to respiratory distress. Data on the perinatal transmission of SARS CoV-2 infection to the fetus are limited. Many studies like Chen et al. and Zhu et al. have suggested no risk of perinatal transmission [16,19].

In a study by Yan et al. [20], there were 86.0% (86/100) of neonates who undergone testing and their pharyngeal swab test for SARS- CoV-2 came out to be negative. Of those 86 neonates, 10 newborns had their cord blood samples and amniotic fluid samples tested; 12 and six delivered females had their breast milk and vaginal secretion sample tested, respectively, and the result of all different samples of patients and their newborn came out to be negative.

Thus, the above statements point us towards the conclusion that there is no proof of intrauterine infection via vertical transmission or the risk of perinatal transmission via breastfeeding as similar findings were reported in the current study. There were two documented IUD cases both due to PIH. One maternal death was reported due to antepartum eclampsia which was unrelated to COVID-19 complications.

Our current study has some limitations. The major drawback is the small sample size due to the pressing need in the pool of lacking data. Second, the antenatal patients in their first and second trimester who were discharged after recovering were lost to follow-up and their data leave a void in the research, and follow-up data for post-recovery complications and late neonatal complications are necessary, and therefore, the current study is still ongoing. However, this article is the preliminary window to determine pregnancy outcomes during the COVID-19 pandemic.

## Conclusions

There are only a few studies that deal with the impact and reverberations of SARS CoV-2 infection on child-bearing females and their newborns and there is not enough evidence to draw a definitive conclusion on its exact potential. In the current study, the spectrum of Coronavirus infection leans more towards asymptomatic and mild symptomatic clinical presentation. Favourably, the likelihood of spontaneous preterm birth was not escalated in our current study and remained low. The rate of intrauterine Fetal Demise and neonatal death were less. As none of the neonates tested positive for COVID-19, there is no corroborative proof of vertical perinatal transmission and as the sample size is too low, it is difficult to draw a definitive conclusion. However, prolonged follow up is required to keep a check on any latent effects on the newborn. As the number of COVID-19 positive patient continues to increase, more studies are needed to be performed on the pregnant patients and their neonates. As the patient pool of only 100 patients is small, the ongoing aggregation of clinical data and research is currently underway with the aim to answer some pressing questions about the spectrum of the disease, risk of congenital infection, optimal intrapartum approach, and the mode and timing of delivery in order to strengthen the COVID-19 protocols.
